# Chronic Pediatric Pain in Low- and Middle-Income Countries

**DOI:** 10.3390/children5090113

**Published:** 2018-08-27

**Authors:** Camila B. Walters, J. Matthew Kynes, Jenna Sobey, Tsitsi Chimhundu-Sithole, K. A. Kelly McQueen

**Affiliations:** 1 Department of Anesthesiology, Vanderbilt University Medical Center, 2200 Children’s Way, Nashville, TN 37209, USA; j.matt.kynes@vanderbilt.edu (J.M.K.); jenna.m.helmer@vanderbilt.edu (J.S.); kelly.mcqueen@vanderbilt.edu (K.A.K.M.); 2 College of Health Sciences, University of Zimbabwe, P.O. Box MP 167, Harare, Zimbabwe; tsitsic98@gmail.com

**Keywords:** chronic pediatric pain, low income country, middle-income country, low- and middle-income country, pediatric palliative care

## Abstract

Chronic pain is a serious health concern and potentially debilitating condition, leading to anxiety, depression, reduced productivity and functionality, and poor quality of life. This condition can be even more detrimental and incapacitating in the pediatric patient population. In low- and middle-income countries (LMICs), pain services are often inadequate or unavailable, leaving most of the world’s pediatric population with chronic pain untreated. Many of these children in LMICs are suffering without treatment, and often die in pain. Awareness and advocacy for this population must be prioritized. We reviewed the available literature on the chronic pediatric pain burden in LMICs, barriers to treatments, and current efforts to treat these patients.

## 1. Introduction

Chronic pain is a serious health concern and potentially debilitating condition, leading to anxiety, depression, reduced productivity and functionality, and poor quality of life [1]. In high income countries (HICs), pediatric chronic pain is treated by specialists in pain clinics or by other physicians whose training has included management of pain, such as anesthesiologists and oncologists. The available services range from a variety of medications, invasive procedures, and psychological and physical therapy. Alternative medicine such as yoga, mindfulness, music therapy, massage, biofeedback and acupuncture are also becoming more frequently utilized in some high resource settings for children with pain [2]. Children with terminal conditions often receive palliative treatment for the underlying condition in the form of surgery or medical treatment. In low- and middle-income countries (LMICs), many of these services are inadequate or unavailable, leaving most of the world’s pediatric population with chronic pain untreated [3]. Many of these children in LMICs are suffering without treatment and often die in pain. Awareness and advocacy for this population must be prioritized.

We performed a review of the relevant literature using the search terms “pediatric or paediatric or children” AND “pain” AND “chronic or persistent” AND “low and middle-income countries (we also listed all of these countries individually)” to compile relevant articles. References within manuscripts were reviewed for further references. Searches were performed through the PubMed, MEDLINE, PsycINFO, Cochrane, African Index Medicus, African Journals Online, and Bioline databases. Although cancer and infectious disease manuscripts were included, care was taken to include manuscripts that described chronic pain secondary to these diseases.

## 2. Review

### 2.1. Pediatric Chronic Pain

Pain is described by the International Association for the Study of Pain (IASP) as “an unpleasant sensory and emotional experience associated with actual or potential tissue damage, or described in terms of such damage” [4]. While acute pain immediately follows an insult or injury, chronic pain is a separate entity that affects an individual’s well-being, is continuous, and recurs beyond the expected normal time of healing or lasts longer than 3–6 months. Pain is further divided into nociceptive pain, pain perceived through specific pain receptors on the surface of somatic tissues or viscera, or neuropathic pain, which is caused by damage to nerves.

Pediatric chronic pain prevalence is estimated to be 20–35% globally [5]. Chronic pain in children is the result of a dynamic integration of biological processes, psychological factors and socio-cultural factors considered within a developmental trajectory. Chronic pain includes persistent (ongoing) and recurrent (episodic) pain in those with chronic health conditions and pain that is the disorder itself, such as migraines, functional abdominal pain or complex regional pain syndrome [5]. Chronic pain in children may be caused by chronic diseases (inflammatory bowel disease, sickle cell, rheumatologic disorders), trauma, life-threatening diseases (cancer, HIV), or may be idiopathic such as headaches [6]. Pediatric chronic pain is complex, and can include nociceptive, neuropathic, affective, sociocultural, behavioral, and cognitive components, and therefore requires multimodal management [7]. Pediatric neuropathic pain has been rarely studied [6]. A systematic review on the epidemiology of chronic pain in children and adolescents found the prevalence rates of pain to be 8–83% for headaches, 4–53% for abdominal pain, 14–24% for back pain, 4–40% for musculoskeletal pain, 4–49% for multiple pains, and 5–88% for other pains. Although the review included mostly high- and very high- income countries, it found that lower socioeconomic status was associated with a higher pain prevalence [8]. Similarly, a Brazilian study found higher dental pain in children of a lower socioeconomic status and lower parental educational levels [9]. 

Children cannot always communicate effectively, but “the inability to communicate verbally does not negate the possibility that an individual is experiencing pain and is in need of appropriate pain-relieving treatment” [10]. The overall experience of pain is not only caused by noxious stimuli, but by cognitive, affective, and behavioral responses. These are affected by social and cultural contexts which we are just beginning to understand [6]. In addition to the physical and emotional distress children may experience while in pain, parental distress over pain management is significant and appropriate pain management leads to increased parental satisfaction [11].

Physical symptoms, psychological distress, and social consequences can severely decrease the quality of life of chronic pain patients. Pain can cause diminished mobility, loss of strength, decreased immune system function, inability to eat, concentrate, sleep, or interact with others [12]. Pain experienced very early in life may have effects on nociceptive perception for the rest of the patient’s lifespan [13]. Children with chronic pain see effects on daily life, including school attendance, family interactions, and social relationships. A Pakistani study found children with chronic dental pain had difficulty sleeping and playing [14]. Another systematic review of pediatric patients reported an association between chronic pain and lower quality of life, as well as presence of other psychological symptoms, such as feeling low, irritable or bad temper, or feeling nervous [8]. Disability related to chronic pain into adulthood can have devastating economic consequences on families and countries that cannot afford to have members unable to work due to pain. 

### 2.2. Pain Treatment Is a Human Right

Pain treatment is considered an obligation under the right to health as a basic human right, as stated by the IASP and the World Health Organization in 2004 [12]. In 2005, the Montreal Statement of Human Right to Essential Medicines stated all have a right to the WHO essential medicines, which includes opioids [15]. The IASP Global Day Against Pain in Children was held in 2005 and focused on pediatric pain. The United Nations Convention of Rights of the Child noted children are a particularly vulnerable part of the population and are therefore entitled to special consideration. In the Declaration of Montreal in 2010, the IASP declared that all people have a right for acknowledgement of their pain and to pain management without discrimination by trained professionals. Failure of government and institutions to provide systems for pain management and failure of health care providers to treat pain within their legal scope of practice was declared to be unethical and a breach of a patient’s human rights [16]. Pain is increasingly accepted as requiring treatment, and under-treatment of pain is recognized as causing short- and long-term negative consequences [17]. The current failure of most governments to appropriately treat pediatric pain is not only a failure to meet an agreed-upon human right, but also a failure to address this vulnerable population.

### 2.3. Lost Disability-Adjusted Life Years (DALYs)

Disability-Adjusted Life Years (DALYs), which include years of life lost from premature death and years lived with disability (YLD), is the most widely used measure of the global burden of disease. Eight out of twelve top disabling non-communicable diseases (NCDs) were related to chronic pain: low back pain, neck pain, migraines, arthritis, other musculoskeletal disorder, depression, anxiety, and drug use disorder [18]. Low back pain was the leading cause of YLDs [19]. Chronic pain syndrome includes multiple somatic complaints with psychological components, such as somatoform disorder or fibromyalgia, and has a prevalence of one to four percent of the global population [20]. Pediatric pain patients have high levels of pain-associated disability [13,21]. Low back, neck pain, and migraines are leading causes of YLD in older children and adolescents [22]. Despite the high disability attributed to migraines, headaches, and musculoskeletal disorder, they have been a low health care priority [22].

### 2.4. Low- and Middle-Income Countries (LMICs)

LMICs are ranked by the World Bank using the Human Development Index (HDI), which averages achievements in three dimensions of human development: a long and healthy life, knowledge, and standard of living. In 2016, low income countries had an average annual gross national income (GNI) of $2649 and middle-income countries of $6281. By comparison, high-income countries’ GNI was $13,844 and very high $39,605 [23]. Disparities between HICs and LMICs are not purely economical, but there are large inequalities in the treatment of disease, including chronic pain.

### 2.5. Chronic Pain Burden in LMICs

The World Health Organization estimates that 5.5 billion people (83% of the world’s population) have no or inadequate treatment for moderate to severe pain, including no access to controlled medicines [16]. Millions of cancer and HIV patients suffer from moderate to severe pain with no treatment, including 1 million end-stage AIDS patients every year, 5.5 million terminally ill cancer patients, and 0.8 million trauma victims [24]. Prevalence of chronic pain varies with gender, age, occupational stress, socioeconomic status, population density, and education [20].

A meta-analysis of the prevalence of chronic pain without a clear etiology in LMICs found the prevalence of unspecified chronic pain to be 34% amongst the general population [25]. A survey of 17 countries found a chronic pain prevalence of 37.3% in developed countries and 41.1% in developing countries. Within the developing countries, there was a wide variability from a 28.4% prevalence in Lebanon to a 58.2% prevalence in Ukraine, potentially showing cultural differences [26]. 

In addition to the high prevalence of pain of 34% in LMICs, 1 in 10 people develop chronic pain every year worldwide [20]. In addition to inequalities between HICs and LMICs, even within LMICs, there is inequality, with the poorest countries carrying the highest burden of chronic pain and decreased availability of treatment [27]. A survey of emergency rooms in central Africa found inadequate pain treatment [28].

### 2.6. Chronic Pediatric Pain Burden in LMICs

While there is paucity of pediatric chronic pain prevalence studies in LMICs available, the Global Burden of Disease 2013 Study found that within the largest 50 countries, there was a 2% prevalence of pediatric low back and neck pain and 5.5% prevalence of migraines for the developing group [22]. At a cancer hospital in Jordan, 48% of pediatric patients had pain, 11% had “a lot” of pain, with only 22% receiving analgesics. Only one child was ordered continuous opioids, all other medications were ordered as needed, showcasing cultural differences from HICs [29]. In Malawi, 27% of children referred for palliative care complained of pain [30]. In Indian children, chronic daily headaches have a prevalence of 6.3% [31] and 10% of school-aged children have chronic abdominal pain [32]. An Indian paper described a chronic pain incidence of 23% in children who presented with a history of abdominal pain [33]. Pelvic pain was present in 37% of adolescent girls in Indian schools and colleges [34]. In a survey of two schools in Sri Lanka, 35.2% of children reported abdominal pain [35]. Colombian children experienced recurrent abdominal pain in 10–15% of school-aged children and up to 75% of teenagers, with 21% with enough pain to affect their daily living. Most cases (85–90%) had no structural basis for the pain and were deemed functional [36]. Five percent of patients in a Colombian study were found to have temporomandibular joint pain [37]. Amongst Nigerian teenagers, low back pain was present in 51.8% in their lifetime, 38.4% in the last 12 months, and 22.3% in the last month. About 41% reported a recurrence of the pain and 13.1% had been absent from school due to the pain [38]. In a cohort of Congolese children, the reported prevalence of odynophagia was 19–21% and nasal pain was 18–20% [39]. In Vietnam, 47.1% of children in kindergarten and preschool had mouth pain, likely secondary to untreated caries [40]. 

Infectious diseases are common in LMICs and can cause chronic pain. In Haiti, 37% of pediatric patients with Tungiasis wounds reported pain. Chronic ear pain was prevalent in 44% of patients with HIV infection in Angola [41]. Twenty percent of pediatric patients with cerebral malaria in Mali reported headaches [42]. Also in Mali, a study looking for schistosomiasis prevalence also found 27.2% of participants had abdominal pain and 23.2% headaches [43]. A large study in Kenya described a 51.1% prevalence of abdominal pain in children with cryptosporidiosis [44]. Children with HIV/AIDS in Colombia had a 19% prevalence of abdominal pain [45]. 

Because children cannot all readily communicate, assessment must be done many times through the observation of behavior and will vary with age, cognitive development, and sociocultural context [6]. Pain assessment scales are in regular use in HICs, but there are few studies addressing the use of pain scales in LMICs. Chronic dental pain prevalence was reported as 25–27% in Nigeria. In this study, the Visual Analog Scale and the Full Cup Test were of similar sensitivity for pain assessments [46]. Indian children in East Delhi assessed their post dental extraction pain as 0.73–4.38/10 using both a visual analog scale and faces pain scale, with decreasing pain with older age. In that setting, the Wong-Baker faces pain rating scale was found to be the more sensitive pain assessment tool [47]. The six-grades faces pain scale was validated in South Africa for postoperative pain in children [48].

Children in LMICs with chronic pain are particularly vulnerable to pain and suffering due to inadequate availability of pain treatment, low personnel capacity with few pain specialists, poor training, cultural differences including language barriers that make it difficult to assess pain, and limited resources [49]. In Malawi, during a 15 month observation period, assessment and management of pediatric pain was not performed [50] and in another review, pain management was not available for pediatric palliative care patients [51]. Nurses in Morocco felt "powerless" because they could not alleviate the suffering of children in pain. Physicians and nurses in Morocco did not feel they had the resources or training to assess and treat pain [52].

### 2.7. Current Efforts in LMICs

Specialist care is rare in LMICs and task shifting is common [53]. When available, specialized care tends to be clustered around cities and is therefore unavailable to rural communities [51]. Most pain conditions are treated by general practitioners, and there are few pain specialists. In Mozambique, an African country with a population of over 25 million people, there are just 2 physicians and 2 nurses specifically trained in pain management. There are 4 pain clinics with 9 anesthesiologists, 1 pediatrician, and 5 rotating resident staff; 3 of these are outside of the capital. The clinic will see pediatric patients, but few children are brought for help, almost all oncology patients. One pain physician saw only 5 pediatric patients in a one-year period. Similarly, in Kenya, anesthesiologists will provide pain care, with few pain specialists in the country. Zimbabwe has only one adult chronic pain specialist and one palliative care specialist, both in the capital city. In Uganda in 2000, new laws allowed specially trained nurses and clinical officers to prescribe morphine to ensure further availability of the drug to those who need it [54].

A systematic review of the availability of pediatric palliative care in LMICs found that 12 out of 19 countries had access to specialized training [51]. When present, the ratios of palliative care service to the population are significantly lower in LMICs than HICS. In Kenya, the ratios are 1:4.28 million and in Pakistan 1:158 million. By comparison, the ratio of services in the United Kingdom is 1:43,000 [55]. Despite most children requiring palliative care services spending a significant amount of time in the hospital, inpatient palliative care is growing at a slow pace. Developed programs are available in Malawi and South Africa. Furthermore, there are only a few pediatric palliative care training opportunities. A multi-disciplinary certificate course for pediatric palliative care has scaled-up training in Tanzania, Uganda and South Africa. This has been in an effort to establish Beacon centers for palliative care for children. Courses have been conducted in Kenya, Zambia, Malawi, and Uganda [56]. In Zimbabwe, the government has formulated the National Strategy for Palliative Care 2020 to address policy, education, and medicine availability for palliative care [57].

### 2.8. Why the Insufficient Efforts?

There are many reasons for the inaccessibility of adequate pain treatment, the paucity of global pain data, and why pain has not been a priority. These reasons include inadequate resource availability and cultural differences. Attitudes towards pain will depend on culture, religion, and political environment, and this will change how pain is perceived and accepted [12,58]. For example, many providers still believe that opioids will cause harm to patients and society due to dependence, which is an unfounded belief for legitimate medical use [12,49]. Nurses in central Africa believed opioids caused addiction, that taking pain medicine was a sign of weakness, and that pain relief could interfere with healing [28]. Some cultures believe showing pain to be weakness, and children in pain may have been taught not to express their concerns [52]. In Malawi, children with significant acute pain such as open fractures were silent, and Kenyan nurses valued stoicism [50]. Parents of pediatric chronic cancer pain patients in Jordan believed that pain was from God and therefore must not be treated. They also believed that children should be responsible for complaining of pain, not asked [29]. These beliefs and attitudes may contribute to the under treatment of pediatric pain.

Global pain is a new field with limited literature and little research. Pain is subjective and currently there are no easy ways to measure physiologic changes due to pain. Even with the pain assessment methods and scales that exist, there is such high heterogeneity that it is difficult to compare them. The reason for this is both the lack of standardization and the lack of necessity, as pain rating scales need to be adapted to local environments. There are large gaps in available pediatric pain research, where most studies have focused on acute pain in children. The WHO has called on the scientific community to research pediatric chronic pain and treatments. In addition to medication research, they call for the development and validation of measurement tools for persistent pain [6]. Standardization of measurement tools and prevalence studies will allow measurements of the current problem of pediatric pain and guide future efforts to alleviate suffering in children.

Because chronic pain is a complex disorder that has physiological, psychological, and cultural components, management is best accomplished with multidisciplinary teams, which are limited in low-resource settings. In LMICs, healthcare workers are often poorly trained with respect to pain. Even in situations where a pain specialist is available, support providers such as physical therapists, psychiatrists, psychologists, and social workers may not be available and treatment will be limited and likely inadequate. Social support, complementary medicine (yoga, acupuncture), and rehabilitation programs may decrease pain and can potentially be helpful with or without needed specialists and medications. Pediatric pain and distress may be reduced with anxiety-reducing skills, education, breathing, muscle relaxation, hypnosis, and imagery [13]. When developing programs to treat pediatric chronic pain, efforts to increase support staff would be a low-cost method to decrease the global burden of pain and its related disability. 

### 2.9. Drug Shortages

The WHO Pain Relief Ladder recommendations for mild to severe pain include different levels of opioids, as well as adjunct pain medications that work in other pain receptor systems. The WHO calls strong opioids such as morphine “absolutely necessary”, and it is recommended as the drug of choice for moderate to severe pain in pediatric patients [6,59]. The weak opioid codeine, morphine, and fentanyl patches are part of the WHO’s List of Essential Medicines [60]. The UN Economic and Social Council have called for adequate opioid availability for medical use and the International Narcotics Control Board is charged with ensuring availability as well as making sure that drugs are not diverted to illicit markets [61]. Although the Single Convention on Narcotics Drugs declared narcotics essential in the treatment of pain four decades ago, most of the world population still does not have access to these medications [62]. There are huge inequalities with access to opioid analgesics, with six developed countries accounting for 79% of the global consumption of morphine, while developing countries, which make up 80% of the world’s population, accounting for 6% [63].

LMICs suffer from drug supply chain problems, with rural hospitals often having minimal, inadequate, or no medications available. Further, there are many restrictions on opioids and essential medicines, making the shortage of opioids even worse than other medications [16]. Due to restrictive regulations, healthcare workers are afraid of legal sanctions for legitimate narcotic use for patients. In some countries, overly restrictive regulations for licit drugs deprive the majority of the population access to opioids when needed for medical reasons [64]. Jordan has restrictive laws for ordering and dispensing opioids, leading to inadequate public access [29]. Where governments have accepted and enacted pain regulation, there is often failure to act on policies. Even when available, pain medication is expensive, with morphine costing over a month’s wage in some LMICs and therefore not accessible to the destitute [12,29,52]. The use of alternative medicines and local healers is common in many LMICs. Complementary medicines were specifically used for pain in Nigeria [65].

A systematic review in LMICs found that only 7 out of 21 countries reported full access to essential analgesic drugs [51]. Two-thirds of the world had little to no access to opioids for pain relief and only 7.5% of the population had adequate access in 2010 [66]. In Africa, 39% of palliative care providers did not have access to opioids [49]. In Malawi, a major hospital only had aspirin readily available for pain treatment, which should not be used in children [49].

The WHO recommends countries prioritize appropriate pain management through comprehensive policies and regulation addressing educational, supply, and cultural barriers. Multidisciplinary teams with both pharmacological and non-pharmacological approaches are recommended to ameliorate chronic pain. Needs assessments with the current burden of pain as well as cost estimates must be done. Pain management ought to be undertaken at every level of the system, from community workers to tertiary care centers. Given that chronic pain has major economic impacts due to patients often being unable to work, countries will save resources by treating pain [6].

### 2.10. Efforts to Improve Access to Chronic Pain Treatment

Current efforts to improve global capacity to appropriately treat chronic pain include framing pain as a human right and appealing to ethical practice and good medicine. Regulatory efforts include negligence and criminal law, elder abuse law, and deregulation of practices that limit opioid availability [12,61]. Guidelines have been created to help countries analyze their own regulatory systems, as well as workshops and seminars [59]. The last decade has seen an increase in scientific literature relating to pain and awareness efforts, starting with the “Global Day Against Pain” in 2004. Guidelines and standards have been created by the IASP, WHO, and other organizations. The WHO recently created guidelines specific to pediatrics, the WHO Guidelines on the pharmacological treatment of persisting pain in children with medical illnesses [6]. These describe the classification of pain, medical evaluation, treatment, and legal information and are available in multiple languages. Efforts to improve the availability of pain medications are underway [12]. 

The IASP has created the Developing Countries Working Group to award grants for educational projects focused on the treatment of pain in LMICs [67]. The World Federation of Societies of Anaesthesiologists has anesthesia-based fellowships in 16 countries spanning 8 specialties. These target young physicians who want to return to their countries and preferably practice in a teaching hospital. Pain fellowships are located in India, Thailand, South Africa, Argentina, and Brazil, with the potential to train 10 fellows per year. Fellowships are as short as 2 months and as long as 1 year, consistent with the American 1-year pain fellowship [68]. Other efforts by the WFSA include information and publications about pain management [69].

Given that most pain management in LMICs is administered by non-anesthetists, the Australia and New Zealand College of Anaesthetists has developed a 3-day training course called Essential Pain Management designed to teach local practitioners to recognize, assess, and treat pain using real-life scenarios. Participants are also given teaching skills to run their own workshops [70,71]. In South Africa, a home-based pediatric palliative care training and support package was developed to equip community workers in rural areas to provide palliative care to very sick children [72]. A Malawi pediatric palliative care team works in both hospital and home settings, and pain control was described as adequate for pediatric oncology patients by authors at the Queen Elizabeth Central Hospital [73]. Other successful pediatric palliative care teams in LMICs have been described, and comprehensive palliative care was achieved in low resource settings, even with no broader systemic support [51,55]. 

Other NGOs and organizations have programs where specific LMIC providers are trained abroad in a higher resource setting. For example, the head of the pain service of Mozambique was invited for a 2-year PhD in palliative care in Portugal as part of a United Nations Educational Scientific and Cultural Organization (UNESCO) initiative. ChildKind is another program that, although not specific to LMICs, hopes to decrease childhood pain worldwide. The program includes a package sent to interested institutions with guidelines, policies, protocols, audit strategies, and educational modules in order to create a pediatric pain management plan. ChildKind principles, including creating evidence-based written policy, assessing all children for pain, and having treatment plans apply to high and low resource settings and the program offers technical expertise to institutions that may help LMIC initiatives [17].

## 3. Conclusions

The last decade has seen increased efforts to decrease the suffering of chronic pain patients with increased awareness, advocacy, and creation of guidelines. There is a paucity of data of pain patients in LMICs, and almost no data on pediatric chronic pain. Billions of children are suffering from chronic pain every year, with personal and national economic impacts. Research into chronic pain in pediatric patients is desperately needed, including defining the prevalence of persistent pain, validation of assessment tools that are culturally appropriate, and best methods of pain relief in different regions. Healthcare is often limited in LMICs, making the most effective manner to integrate palliative care being through existing services for children. Governments must be encouraged to address drug supply issues and legislation that prevents chronic pain patients from receiving needed medications. Practitioners must be trained in the recognition, assessment, and proper treatment of chronic pain conditions. Education is also warranted in settings where culture prevents proper chronic pain diagnosis and treatment. In low resource settings, a focus on creation of multidisciplinary teams that can develop low cost pain treatment strategies with nonpharmacological therapies, such as physical therapy and psychological support, may be warranted. These efforts will vary by country and must be tailored to local culture and resources. The work to decrease chronic pediatric pain suffering is only beginning.

## References

[1] Gureje O., Von Korff M., Simon G.E., Gater R. (1998). Persistent pain and well-being: A World Health Organization study in primary care. JAMA.

[2] Lin Y.C., Lee A.C., Kemper K.J., Berde C.B. (2005). Use of complementary and alternative medicine in pediatric pain management service: A survey. Pain Med..

[3] Goucke C.R., Chaudakshetrin P. (2018). Pain: A neglected problem in the low-resource setting. Anesth. Analg..

[4] Loeser J.D., Treede R.D. (2008). The Kyoto protocol of IASP basic pain terminology. Pain.

[5] Palermo T., Eccleston C., Goldschneider K., McGinn K.L., Sethna N., Schechter N., Turner H. Assessment and Management of Children with Chronic Pain. A Position Statement from the American Pain Society. http://americanpainsociety.org/uploads/about/position-statements/Pediatric%20Pain%20Policy.pdf.

[6] World Health Organization (WHO) (2012). Guidelines on the Pharmacological Treatment of Persisting Pain in Children with Medical Illnesses.

[7] Liossi C., Howard R.F. (2016). Pediatric chronic pain: Biopsychosocial assessment and formulation. Pediatrics.

[8] King S., Chambers C.T., Huguet A., MacNevin R.C., McGrath P.J., Parker L., MacDonald A.J. (2011). The epidemiology of chronic pain in children and adolescents revisited: A systematic review. Pain.

[9] Nomura L., Bastos J., Peres M. (2004). Dental pain prevalence and association with dental caries and socioeconomic status in school children. Braz. Oral Res..

[10] Merskey H., Bogduk N. (1994). Classification of Chronic Pain: Descriptions of Chronic Pain Syndromes and Definitions of Pain Terms.

[11] Ammentorp J., Mainz J., Sabroe S. (2005). Parents’ priorities and satisfaction with acute pediatric care. Arch. Pediatr. Adolesc. Med..

[12] Brennan F., Carr D.B., Cousins M. (2007). Pain management: A fundamental human right. Anesth. Analg..

[13] International Association for the Study of Pain (2005). Why children’s pain matters. Pain.

[14] Pau A., Khan S.S., Babar M.G., Croucher R. (2008). Dental pain and care-seeking in 11–14-yr-old adolescents in a low-income country. Eur. J. Oral Sci..

[15] Pogge T. (2007). Montreal statement on the human right to essential medicines. Camb. Q. Health Ethics.

[16] International Pain Summit of the International Association for the Study of Pain (2011). Declaration of Montreal: Declaration that access to pain management is a fundamental human right. J. Pain Palliat. Care Pharmacother..

[17] Schechter N., Finley G., Bright N., Laycock M., Forgeron P. (2010). ChildKind: A global initiative to reduce pain in children. Pediatr. Pain Lett..

[18] Buchbinder R., Blyth F.M., March L.M., Brooks P., Woolf A.D., Hoy D.G. (2013). Placing the global burden of low back pain in context. Best Pract. Res. Clin. Rheumatol..

[19] Hoy D., March L., Brooks P., Blyth F., Woolf A., Bain C., Williams G., Smith E., Vos T., Barendregt J. (2014). The global burden of low back pain: Estimates from the Global Burden of Disease 2010 study. Ann. Rheum. Dis..

[20] Jackson T., Stabile V., McQueen K. (2014). The global burden of chronic pain. ASA Newsl..

[21] Bursch B., Walco G.A., Zeltzer L. (1998). Clinical assessment and management of chronic pain and pain-associated disability syndrome. J. Dev. Behav. Pediatr..

[22] Kyu H.H., Pinho C., Brown J.C., Wagner J.A., Bertozzi-Villa A., Charlson F.J., Coffeng L.E., Dandona L., Erskine H.E., Global Burden of Disease Pediatrics Collaboration (2016). Global and national burden of diseases and injuries among children and adolescents between 1990 and 2013: Findings from the global burden of disease 2013 study. JAMA Pediatr..

[23] (2016). Human Development Report 2016.

[24] World Health Organization (WHO) (2012). Briefing Note: Access to Controlled Medications Programme.

[25] Jackson T., Thomas S., Stabile V., Shotwell M., Han X., McQueen K. (2016). A systematic review and meta-analysis of the global burden of chronic pain without clear etiology in low- and middle-income countries: Trends in heterogeneous data and a proposal for new assessment methods. Anesth. Analg..

[26] Tsang A., Von Korff M., Lee S., Alonso J., Karam E., Angermeyer M.C., Borges G.L., Bromet E.J., Demytteneare K., De Girolamo G. (2008). Common chronic pain conditions in developed and developing countries: gender and age differences and comorbidity with depression-anxiety disorders. J. Pain.

[27] Goldberg D.S., McGee S.J. (2011). Pain as a global public health priority. BMC Public Health.

[28] Rampanjato R.M., Florence M., Patrick N.C., Finucane B.T. (2007). Factors influencing pain management by nurses in emergency departments in Central Africa. Emerg. Med. J..

[29] Forgeron P.A., Finley G.A., Arnout M. (2006). Pediatric pain prevalence and parents’ attitudes at a cancer hospital in Jordan. J. Pain Symptom Manag..

[30] Lavy V. (2007). Presenting symptoms and signs in children referred for palliative care in Malawi. Palliat. Med..

[31] Chakravarty A. (2005). Chronic daily headache in children and adolescents: A clinic based study from India. Cephalalgia.

[32] Ganesh R., Arvind Kumar R., Suresh N., Sathiyasekeran M. (2010). Chronic abdominal pain in children. Natl. Med. J. India.

[33] Oak S.N., Parelkar S.V., Akhtar T., Joshi M., Pathak R., Viswanath N.K., Satish Kumar V., Ravikiran K., Manjunath L., Ahmed A. (2005). Minimal access surgery in children—5 years institutional experience. J. Minim. Access Surg..

[34] Wadhwa L., Sharma J.B., Arora R., Malhotra M., Sharma S. (2004). Severity, affect, family and environment (SAFE) approach to evaluate chronic pelvic pain in adolescent girls. Indian J. Med. Sci..

[35] Rajindrajith S., Devanarayana N.M., Adhikari C., Pannala W., Benninga M.A. (2012). Constipation in children: an epidemiological study in Sri Lanka using Rome III criteria. Arch. Dis. Child..

[36] De Vivero R. (2005). Dolor Abdominal Recurrente. Colomb. Med..

[37] Soto L., Hernandez J., Villavivencio J. (2001). Trastornos de la articulación temporomandibular en escolares de 5 a 14 años de un centro educativo de Cali. Colomb. Med..

[38] Adegoke B.O.A., Odole A.C., Adeyinka A.A. (2015). Adolescent low back pain among secondary school students in Ibadan, Nigeria. Afr. Health Sci..

[39] Gedikondele J.S., Longo-Mbenza B., Nzanza J.M., Luila E.L., Reddy P., Buso D. (2011). Nose and throat complications associated with passive smoking among Congolese school children. Afr. Health Sci..

[40] Khanh L.N., Ivey S.L., Sokal-Gutierrez K., Barkan H., Ngo K.M., Hoang H.T., Vuong I., Thai N. (2015). Early childhood caries, mouth pain, and nutritional threats in Vietnam. Am. J. Public Health.

[41] Taipale A., Pelkonen T., Taipale M., Roine I., Bernardino L., Peltola H., Pitkäranta A. (2011). Otorhinolaryngological findings and hearing in HIV-positive and HIV-negative children in a developing country. Eur. Arch. Otorhinolaryngol..

[42] Ngoungou E.B., Poudiougou B., Dulac O., Dicko A., Boncoeur M.P., Traore A.M., Coulibaly D., Keita M.M., Preux P.M., Doumbo O.K. (2007). Persistent neurological sequelae due to cerebral malaria in a cohort of children from Mali. Rev. Neurol..

[43] Tandina F., Doumbo S.N., Kone A.K., Guindo D., Goita S., Sissoko M., Konaté S., Dabo A., Doumbo O.K. (2016). Epidemiology of schistosomiasis in the periurban area of Sotuba, 10 years mass treatment began in Mali. Med. Sante Trop..

[44] Gatei W., Wamae C.N., Mbae C., Waruru A., Mulinge E., Waithera T., Gatika S.M., Kamwati S.K., Revathi G., Hart C.A. (2006). Cryptosporidiosis: Prevalence, genotype analysis, and symptoms associated with infections in children in Kenya. Am. J. Trop. Med. Hyg..

[45] Velasco C.A., Mendez F., Lopez P. (2011). Cryptosporidiosis in Colombian children with HIV/AIDS infection. Colomb. Med..

[46] Odai E., Ehizele A., Enabulele J. (2015). Assessment of pain among a group of Nigerian dental patients. BMC Res. Notes.

[47] Khatri A., Kalra N. (2012). A comparison of two pain scales in the assessment of dental pain in East Delhi children. ISRN Dent..

[48] Bosenberg A., Thomas J., Lopez T., Kokinsky E., Larsson L.E. (2003). Validation of a six-graded faces scale for evaluation of postoperative pain in children. Paediatr. Anaesth..

[49] Albertyn R., Rode H., Millar A.J., Thomas J. (2009). Challenges associated with paediatric pain management in Sub-Saharan Africa. Int. J. Surg..

[50] Walters M. (2009). Pain assessment in Sub-Saharan Africa. Pediatr. Pain Lett..

[51] Caruso Brown A.E., Howard S.C., Baker J.N., Ribeiro R.C., Lam C.G. (2014). Reported availability and gaps of pediatric palliative care in low- and middle-income countries: A systematic review of published data. J. Palliat. Med..

[52] MacCarthy P., Chammas G., Wilimas J., Alaoui F., Harif M. (2004). Managing children’s cancer pain in Morocco. J. Nursing Scholarsh..

[53] Seidman G., Atun R. (2017). Does task shifting yield cost savings and improve efficiency for health systems? A systematic review of evidence from low-income and middle-income countries. Hum. Resour. Health.

[54] Jagwe J., Merriman A. (2007). Uganda: Delivering analgesia in rural Africa: Opioid availability and nurse prescribing. J. Pain Symptom Manag..

[55] Wright M., Wood J., Lynch T., Clark D. (2008). Mapping levels of palliative care development: A global view. J. Pain Symptom Manag..

[56] Guidelines for Using the APCA African Children’s Palliative Outcome Scale. African Palliative Care Association 2012. http://aidsfree.usaid.gov/sites/default/files/pos_guidelines_for_children.pdf.

[57] (2018). Palliative Care Strategic Framework and Integration Roadmap. Integrating Palliative Care in Zimbabwe’s Health System.

[58] Junkins S., Goloshchapov D., Sindt J. (2017). Pain in low resource environments. Curr. Anesthesiol. Rep..

[59] World Health Organization (WHO) (2000). Achieving Balance in National Opioids Control Policy—Guidelines for Assessment.

[60] World Health Organization (2017). WHO Model List of Essential Medicines. http://www.who.int/medicines/publications/essentialmedicines/20th_EML2017_FINAL_amendedAug2017.pdf?ua=1.

[61] Lohman D., Schleifer R., Amon J.J. (2010). Access to pain treatment as a human right. BMC Med..

[62] United Nations (1961). Single Convention on Narcotic Drugs. http://www.incb.org/incb/convention_1961.html.

[63] International Narcotics Control Board (2005). The Report of the International Narcotics Control Board for 2004.

[64] International Narcotics Control Board, United Nations, New York, 2011 Report of the International Narcotics Control Board on the Availability of Internationally Controlled Drugs Ensuring Adequate Access for Medical and Scientific Purposes. http://search.ebscohost.com/login.aspx?direct=true&scope=site&db=nlebk,e000xna&db=nlabk&AN=387607.

[65] Oshikoya K.A., Senbanjo I.O., Njokanma O.F., Soipe A. (2008). Use of complementary and alternative medicines for children with chronic health conditions in Lagos, Nigeria. BMC Complement. Altern. Med..

[66] Duthey B., Scholten W. (2014). Adequacy of opioid analgesic consumption at country, global, and regional levels in 2010, its relationship with development level, and changes compared with 2006. J. Pain Symptom Manag..

[67] Bond M. (2011). Pain education issues in developing countries and responses to them by the International Association for the Study of Pain. Pain Res. Manag..

[68] World Federation of Societies of Anaesthesiologists WFSA Fellowship Programmes. https://www.wfsahq.org/wfsa-fellowship-programmes.

[69] Charlton E. (1997). The management of postoperative pain. Updat. Anaesth..

[70] Evans F.M., Duarte J.C., Haylock Loor C., Morriss W. (2018). Are short subspecialty courses the educational answer?. Anesth. Analg..

[71] Australian and New Zealand College of Anaesthetists Essential Pain Management. http://fpm.anzca.edu.au/fellows/essential-pain-management.

[72] Naicker S.N., Richter L., Stein A., Campbell L., Marston J. (2016). Development and pilot evaluation of a home-based palliative care training and support package for young children in southern Africa. BMC Palliat. Care.

[73] Israels T., Banda K., Molyneux E.M. (2008). Paediatric oncology in the Queen Elizabeth Central Hospital, Blantyre. Malawi Med. J..

